# Quantification of Xanthone and Anthocyanin in Mangosteen Peel by UPLC-MS/MS and Preparation of Nanoemulsions for Studying Their Inhibition Effects on Liver Cancer Cells

**DOI:** 10.3390/ijms24043934

**Published:** 2023-02-15

**Authors:** Rui Li, Baskaran Stephen Inbaraj, Bing-Huei Chen

**Affiliations:** Department of Food Science, Fu Jen Catholic University, New Taipei City 242062, Taiwan

**Keywords:** mangosteen peel, xanthone, anthocyanin, nanoemulsion, UPLC-MS/MS, liver cancer cells HepG2

## Abstract

Mangosteen peel, a waste produced during mangosteen processing, has been reported to be rich in xanthone and anthocyanin, both of which possess vital biological activities such as anti-cancer properties. The objectives of this study were to analyze various xanthones and anthocyanins in mangosteen peel by UPLC-MS/MS for the subsequent preparation of both xanthone and anthocyanin nanoemulsions to study their inhibition effects on liver cancer cells HepG2. Results showed that methanol was the optimal solvent for the extraction of xanthones and anthocyanins, with a total amount of 68,543.39 and 2909.57 μg/g, respectively. A total of seven xanthones, including garcinone C (513.06 μg/g), garcinone D (469.82 μg/g), γ-mangostin (11,100.72 μg/g), 8-desoxygartanin (1490.61 μg/g), gartanin (2398.96 μg/g), α-mangostin (51,062.21 μg/g) and β-mangostin (1508.01 μg/g), as well as two anthocyanins including cyanidin-3-sophoroside (2889.95 μg/g) and cyanidin-3-glucoside (19.72 μg/g), were present in mangosteen peel. The xanthone nanoemulsion was prepared by mixing an appropriate portion of soybean oil, CITREM, Tween 80 and deionized water, while the anthocyanin nanoemulsion composed of soybean oil, ethanol, PEG400, lecithin, Tween 80, glycerol and deionized water was prepared as well. The mean particle size of the xanthone extract and nanoemulsion were, respectively, 22.1 and 14.0 nm as determined by DLS, while the zeta potential was −87.7 and −61.5 mV. Comparatively, xanthone nanoemulsion was more effective than xanthone extract in inhibiting the growth of HepG2 cells, with the IC_50_ being 5.78 μg/mL for the former and 6.23 μg/mL for the latter. However, the anthocyanin nanoemulsion failed to inhibit growth of HepG2 cells. Cell cycle analysis revealed that the proportion of the sub-G1 phase followed a dose-dependent increase, while that of the G0/G1 phase showed a dose-dependent decline for both xanthone extracts and nanoemulsions, with the cell cycle being possibly arrested at the S phase. The proportion of late apoptosis cells also followed a dose-dependent rise for both xanthone extracts and nanoemulsions, with the latter resulting in a much higher proportion at the same dose. Similarly, the activities of caspase-3, caspase-8 and caspase-9 followed a dose-dependent increase for both xanthone extracts and nanoemulsions, with the latter exhibiting a higher activity at the same dose. Collectively, xanthone nanoemulsion was more effective than xanthone extract in inhibiting the growth of HepG2 cells. Further research is needed to study the anti-tumor effect in vivo.

## 1. Introduction

Mangosteen (*Garcinia mangostana* L.), a popular tropical fruit mainly grown in southeast Asian countries such as Thailand, Indonesia and Malaysia, is rich in nutrients and phytochemicals such as xanthones and anthocyanins [1,2]. However, the edible portion (pulp) only accounts for 31% of the whole mangosteen fruit, with the remaining inedible portion being peel (69%). Nevertheless, compared to the pulp portion, the peel portion was shown to contain a 10-fold higher level of polyphenol compounds such as xanthones, anthocyanins and tannins, as well as 20-fold higher antioxidant activity [2]. Thus, it will be a great advantage to the food industry if the polyphenol compounds in mangosteen peel can be isolated and processed into health foods.

Malaysia, a major country producing mangosteen, developed mangosteen maturity indices to satisfy needs of different markets for export. For instance, the fruit is suitable for export when the color is red or brownish red and the separation of the rind and pulp is easy, while the fruit is suitable for consumption when the color is red-purple and no gum is present in the rind [3]. Furthermore, Palapol et al. [4] analyzed the anthocyanin content in mangosteen peel during ripening and reported a higher content of anthocyanin in purple-brown peel than in pink peel by 20-fold. Thus, the peel color change of mangosteen should be monitored carefully to obtain the maximum level of polyphenol compounds for subsequent processing.

In recent years, the biological activities of mangosteen peel, including anti-oxidation, anti-cancer, anti-inflammation, anti-diabetes and cardiovascular protection, have been well documented [5], which can be attributed to the presence of xanthone and anthocyanin. Xanthone, composed of tricyclic aromatic hydrocarbons, possesses high stability and is the most abundant bioactive compound in mangosteen peel. More than 60 xanthones have been characterized in mangosteen, and a much higher content of xanthone was shown in the peel than in pulp, with the main xanthones in the former including α-mangostin, γ-mangostin, 8-desoxygartanin, gartanin, garcinone C, garcinone D and β-mangostin [5,6]. Of the various xanthones in mangosteen peel, α-mangostin was present in the highest amount and investigated most often for its physiological activities. For instance, α- mangostin was shown to possess high antioxidant activity through scavenging singlet oxygen, superoxide anions and peroxynitrite anions [7]. Additionally, both α-mangostin and γ-mangostin were effective in anti-inflammation through a decline of cytokine expressions such as TNF-α, IL-6 and IL-1β [8] and anti-diabetes, through the inhibition of α-glucosidase activity [9]. For an anti-cancer study, α-mangostin was efficient in inhibiting the growth of breast cancer cells (MCF-7) and cervical cancer stem cells, with the former through the decreased expression of MMP-2 and MMP-9, resulting in retarding 12-O-tetradecanoylphorbol-13-acetate (TPA) induced cancer cell metastasis [10], and the latter through the upregulation of Bax and downregulation of Mcl-1 and Bcl-2 for the elevation of caspase-9 and caspase-3 activities, leading to cell apoptosis [11].

Like xanthone, anthocyanin also represents a vital biological compound in mangosteen peel, with cyanidin-3-sophoroside and cyanidin-3-glucoside dominating, and the contents were reported to be 3 and 0.1 mg/g, respectively, when the maturation reached the final stage [12]. However, anthocyanins are highly unstable and susceptible to degradation loss and color change under high pH, high temperature and illumination [13]. In a study dealing with the degradation kinetics of anthocyanins from European cranberry bush fruit extracts, Moldovan et al. [14] reported that the lower the pH, the higher the stability of anthocyanin, and the most optimal pH for the storage of cranberry juice should be controlled at pH 1–3. Khoo et al. [15] further reported that, compared to heating temperature at 25 °C, the grape anthocyanin content was reduced by half when heated at 35 °C, while the blackberry anthocyanin degradation was shown to fit the second-order reaction kinetics, with the half-life being 28.20 h under illumination intensity of 3968.30 lx [16]. Nevertheless, anthocyanins have been reported to exhibit many biological activities. For example, the roselle anthocyanins were shown to possess strong antioxidant activity with the IC_50_ of inhibiting DPPH and ABTS free radicals being 4.06 and 3.7 mg/mL, respectively [17]. In a later study, Lee et al. [18] reported that cyanidin chloride was effective in inhibiting the growth of colon cancer cells including HCT116, HT29 and SW620 through the retardation of NF-kB signaling and the activation of Nrf2. Similarly, the anthocyanin extract prepared from dark sweet cherry was efficient in inhibiting the growth of breast cancer cells, including BT4747, MDA-MB-453 and MDA-MB-231 through the downregulation of the Akt/mTOR route and Sirtl/survivin route [19]. Additionally, during atheroscelosis, anthocyanins were able to minimize the accumulation of low-density lipoprotein in arterial walls and the formation of foam cells, and thus inhibit the atherosclerotic plaque formation [20].

Due to inadequate bioavailability of both xanthones and anthocyanins in vivo, it is necessary to employ appropriate encapsulation techniques such as nanoemulsion to elevate the bioavailability, and thus the biological activity can be greatly enhanced. The advantages of nanoemulsion (10–100 nm) application in food and drug industries have been well documented [21]: 1. the elevation of bioavailability of bioactive compounds; 2. the enhanced solubility of lipophilic bioactive compounds; 3. the rapid and efficient penetration of bioactive compounds into body; 4. the prevention of hydrolysis and the oxidation of bioactive compounds; 5. the masking of undesirable smells; 6. being a carrier of both lipophilic and hydrophilic compounds; 7. the elevation of therapeutic efficiency and the reduction of dosage intake so that the side effect can be minimized; and 8. the elevation of the stability of bioactive compounds in vivo. In addition, the incorporation of the targeting ligand into a nanoemulsion system encapsulated with bioactive compounds can effectively inhibit cancer cells by selective accumulation on tumor sites through the active targeting of receptors overexpressed in cancer cells. In line with this hypothesis, Rosso et al. [22] prepared glutaraldehyde-crosslinked hyaluronic acid submicron particles (200–400 nm) by a non-solvent emulsion method and demonstrated their effective uptake by prostate cancer cells LNCaP without any significant toxicity and high accumulation of hyaluronic acid on a tumor site in a xenograft mice model through active targeting with CD44, a 85 kDa transmembrane receptor overexpressed in most tumor cells. Moreover, a bioactives-encapsulated nanoemulsion system conjugated with magnetic nanoparticles can be successfully used for both therapeutic and imaging applications. For instance, Vecchione et al. [23] reported that an oil-in-water nanoemulsion loaded with curcumin and magnetic cobalt ferrite oxide nanocubes could not only efficiently inhibit melanoma cancer cells B16-F10, but also be successfully applied for photoacoustic and magnetic resonance dual imaging.

Cancer is a leading cause of death worldwide, accounting for nearly 10 million deaths in 2020 or nearly one in six deaths, with the most common cancers being breast, lung, colon and rectum, and prostate cancers [24]. However, in Taiwan, according to a report by the Ministry of Health and Welfare (MOHW) [25], liver cancer ranks as the second cause of cancer death in Taiwan, with about 13,000 deaths from chronic liver disease, liver cirrhosis, and liver cancer. Based on a report by the National Cancer Institute (NCI) of the USA [26], liver cancer can be divided into primary liver cancer and secondary liver cancer, with the former further divided into hepatocellular carcinoma (HCC), intrahepatic cholangiocarcinoma, angiosarcoma, hemangiosarcoma, and hepatoblastoma, in which HCC is the most prevalent type of liver cancer in adults. Specifically, when hepatocytes, the main liver cells, are subjected to harmful conditions (hepatitis virus infections), it can cause hepatitis and lead to cirrhosis and ultimately to HCC [27]. The current therapies for HCC include surgery, liver transplantation, chemotherapy and immunotherapy [27]. However, tumor relapse and metastasis may occur after resection. Thus, the development of a new treatment method of liver cancer such as targeted therapy is important.

The objectives of this study were to extract xanthones and anthocyanins from mangosteen peel for their identification and quantitation by UPLC-MS/MS. Additionally, both xanthone and anthocyanin nanoemulsions were prepared to study and compare their inhibition effects on liver cancer cells HepG2, with the possible mechanism being explored.

## 2. Results and Discussion

### 2.1. Analysis of Xanthones and Anthocyanins in Mangosteen Peel by UPLC-MS/MS

**Figure 1:**
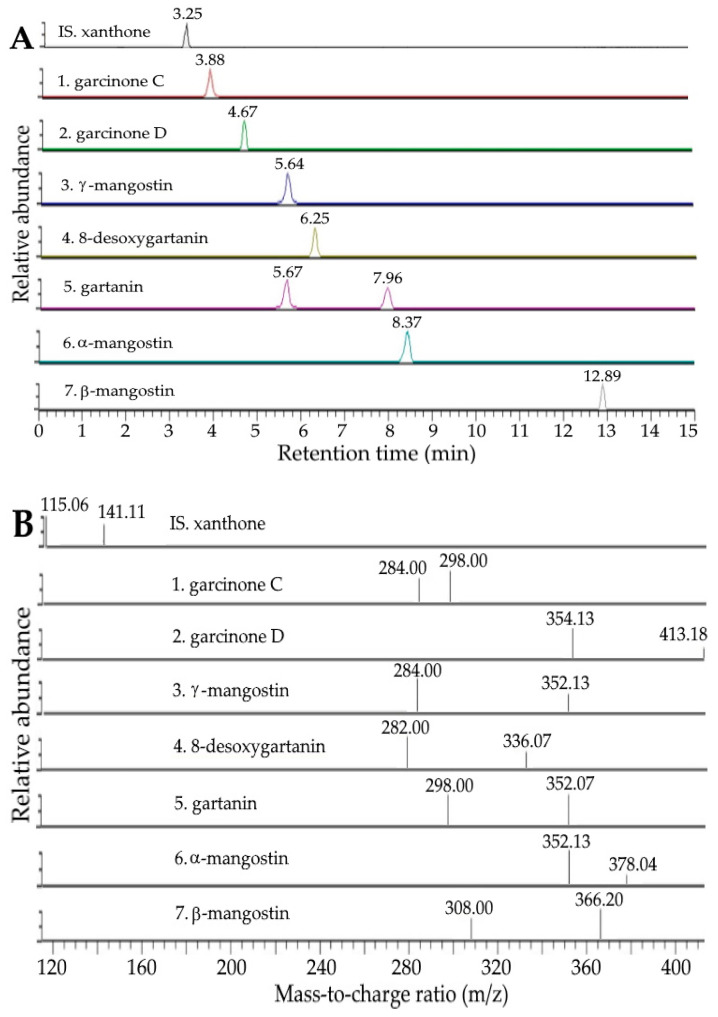
UPLC-MS/MS chromatograms of xanthone standards and internal standard (xanthone) as detected by SRM mode (**A**) and their corresponding tandem mass spectra (**B**).

**Figure 2:**
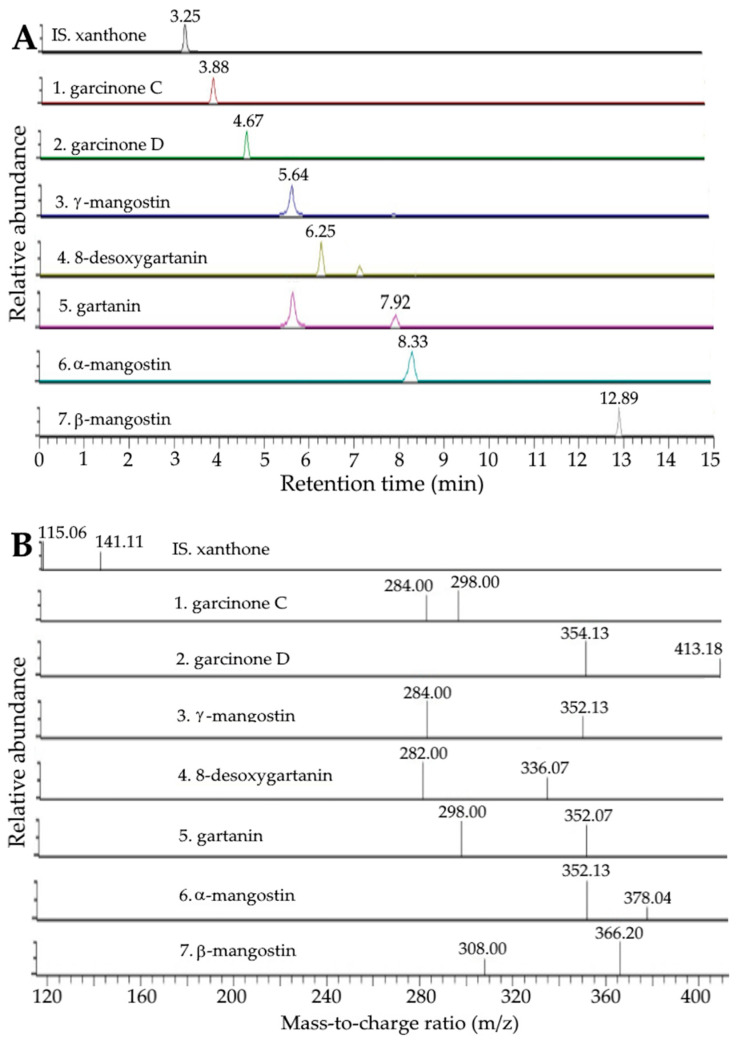
UPLC-MS/MS chromatograms of xanthone extract containing internal standard (xanthone) as detected by SRM mode (**A**) and their corresponding tandem mass spectra (**B**).

**Figure 3:**
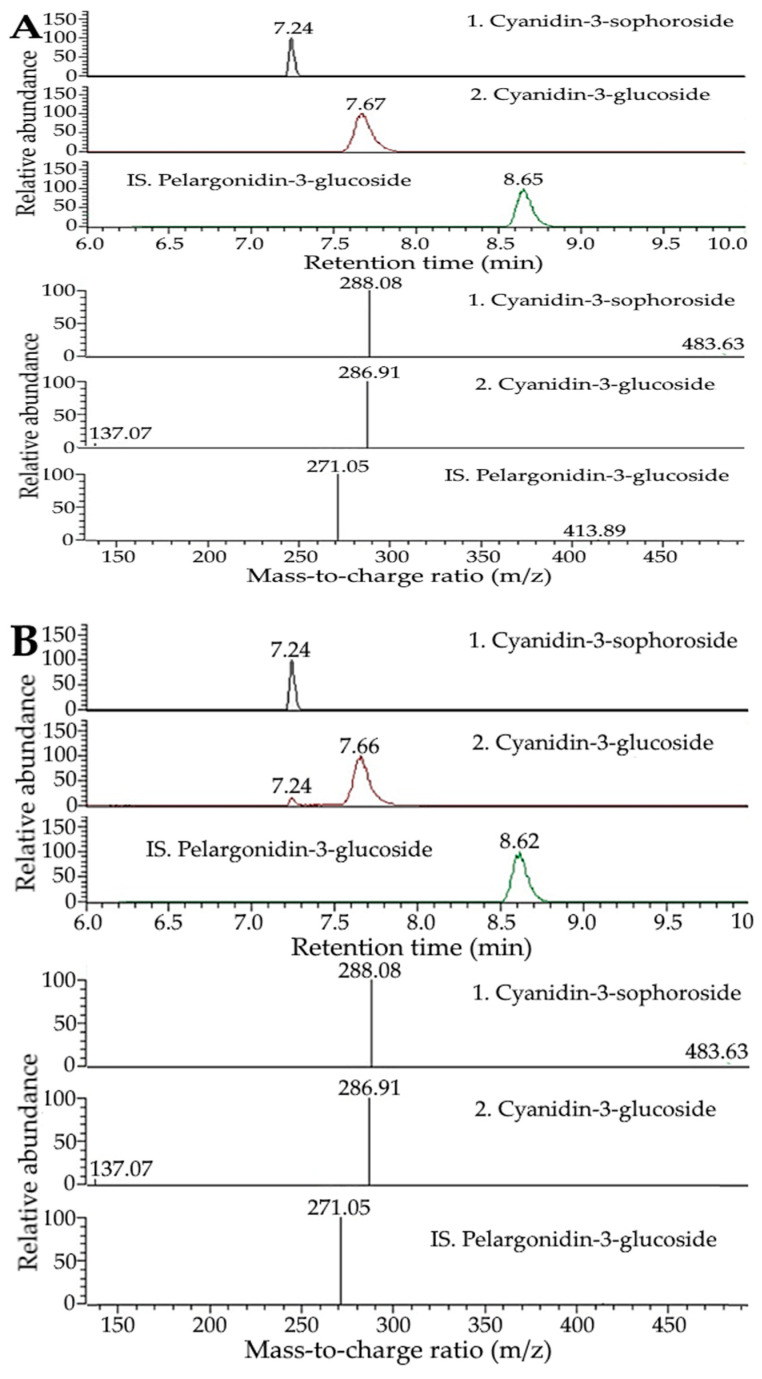
UPLC-MS/MS chromatograms of anthocyanin standards and internal standard perlargonidin-3-glucoside (**A**) and extract containing internal standard perlargonidin-3-glucoside (**B**) as detected by SRM mode.

The UPLC-MS/MS chromatograms of various xanthone standards and internal standard (xanthone) as well as xanthone extracts containing the internal standard are shown in Figure 1 and Figure 2, respectively, while the UPLC-MS/MS chromatograms of anthocyanin standards and internal standard (pelargonidin 3-glucoside), as well as anthocyanin extracts containing the internal standard, are shown in Figure 3A and Figure 3B, respectively. Following the separation criteria as mentioned in the methods section, a total of seven xanthones including garcinone C, garcinone D, γ-mangostin, 8-desoxygartanin, gartanin, α-mangostin and β-mangostin were separated within 13 min, with the retention times being 3.88, 4.67, 5.64, 6.25, 7.96, 8.37, and 12.89 min, respectively (Table 1), while two anthocyanins, including cyanidin-3-sophoroside and cyanidin-3-glucoside, were separated within 8 min with the retention times being 7.25 and 7.67 min, respectively (Table 1). Through the comparison of mass-to-charge ratios (m/z) of both precursor ions and product ions of unknown peaks with standards and those reported in the literature [28,29,30], a total of seven xanthones and two anthocyanins in mangosteen peels were positively identified (Table 1).

Table 1 also shows the contents of xanthones and anthocyanins in dried mangosteen peels extracted by methanol and ethanol. Comparatively, no significant difference (*p* > 0.05) in the total amount of xanthones was shown between methanol and ethanol, while a significantly higher content of total anthocyanins (*p* < 0.05) was found with methanol as the extraction solvent. For quantitation, a total of nine standard calibration curves including garcinone C, garcinone D, γ-mangostin, 8-desoxygartanin, gartanin, α-mangostin, β-mangostin, cyanidin 3-sophoroside and cyanidin 3-glucoside were prepared, with the linear regression equations being y = 0.00861478x − 0.0162142, y = 0.0206076x + 0.030393, y = 0.0138917x + 0.0235967, y = 0.0030961x + 0.00548724, y = 0.00237788x + 0.00480259, y = 0.026519x + 0.0020046, y = 0.00854577x + 0.0191585, y = 0.8167x − 0.0108 and y = 1.5497x − 0.0036, respectively, with R^2^ of all being higher than 0.99. Of the various xanthones, α-mangostin was present in the largest amount (51,062.21 μg/g), followed by γ-mangostin (11,100.72 μg/g), gartanin (2398.96 μg/g), β-mangostin (1508.01 μg/g), 8-desoxygartanin (1490.61 μg/g), garcinone C (513.06 μg/g), and garcinone D (469.82 μg/g), while for anthocyanins, cyanidin-3-sophoroside (2889.95 μg/g) was present at a much higher level than cyanidin-3-glucoside (19.72 μg/g). Thus, methanol was selected as the extraction solvent for subsequent nanoemulsion preparation and cell culture experiments. 

In several previous studies, both α-mangostin and γ-mangostin were also reported to dominate in mangostin peels. For instance, Walker [6] reported the presence of α-mangostin and γ-mangostin at 5.51 × 10^4^ and 1.5 × 10^4^ μg/g, respectively, in dried mangosteen peels produced by Xango Co in USA with 80% acetone as the extraction solvent. In another study with ethyl acetate as the extraction solvent, the contents of α-mangostin and γ-mangostin were, respectively, 47.04 and 6.35 mg/g in dried mangostin peels produced in the eastern Thailand [1], while with methylene chloride as the extraction solvent the levels of α-mangostin and γ-mangostin were 3323.88 and 860.17 mg/100 g in dried mangostin peels produced in Thailand and exported to Germany [29]. However, for anthocyanins, the cyanidin-3-sophoroside content was reported to be 1403 and 3126 mg/kg, respectively, at the 5th and 6th maturation stage [4]. Apparently, the difference in xanthone and anthocyanin contents from various mangostin peels can be attributed to variation in the growth location, harvest time, drying method, extraction solvent variety, and maturation stage.

### 2.2. Method Validation

For method validation, the LODs of garcinone C, garcinone D, γ-mangostin, 8-desoxygartanin, gartanin, α-mangostin, β-mangostin, cyanidin-3-sophoroside and cyanidin-3-glucoside were, respectively, 0.486, 0.393, 0.543, 0.365, 0.408, 0.674, 0.437, 0.0546 and 0.0219 μg/mL, while the LOQs were 1.457, 1.179, 1.629, 1.095, 1.224, 2.022, 1.311, 0.1638 and 0.0657 μg/mL. The mean recoveries of xanthones and anthocyanins in mangosteen peels including garcinone C, garcinone D, γ-mangostin, 8-desoxygartanin, gartanin, α-mangostin, β-mangostin, cyanidin-3-sophoroside and cyanidin-3-glucoside were, respectively, 91.64, 95.93, 96.68, 97.66, 93.98, 96.54, 90.34, 93.15 and 88.37% (Table 2), while the RSDs of the intra-day variability were 2.53, 1.33, 2.87, 1.14, 2.62, 2.19, 0.93, 3.43 and 2.94%, as well as 3.67, 1.99, 2.08, 1.60, 1.56, 2.27, 1.84, 3.70 and 2.60% for the inter-day variability (Table 2). All the recovery data and RSD of the intra-day and inter-day variability data meet the method validation requirement issued by TFDA [31], implying that a high accuracy and precision was established for the method developed in this study.

### 2.3. Preparation and Characterization of Xanthone and Anthocyanin Nanoemulsions

**Figure 4:**
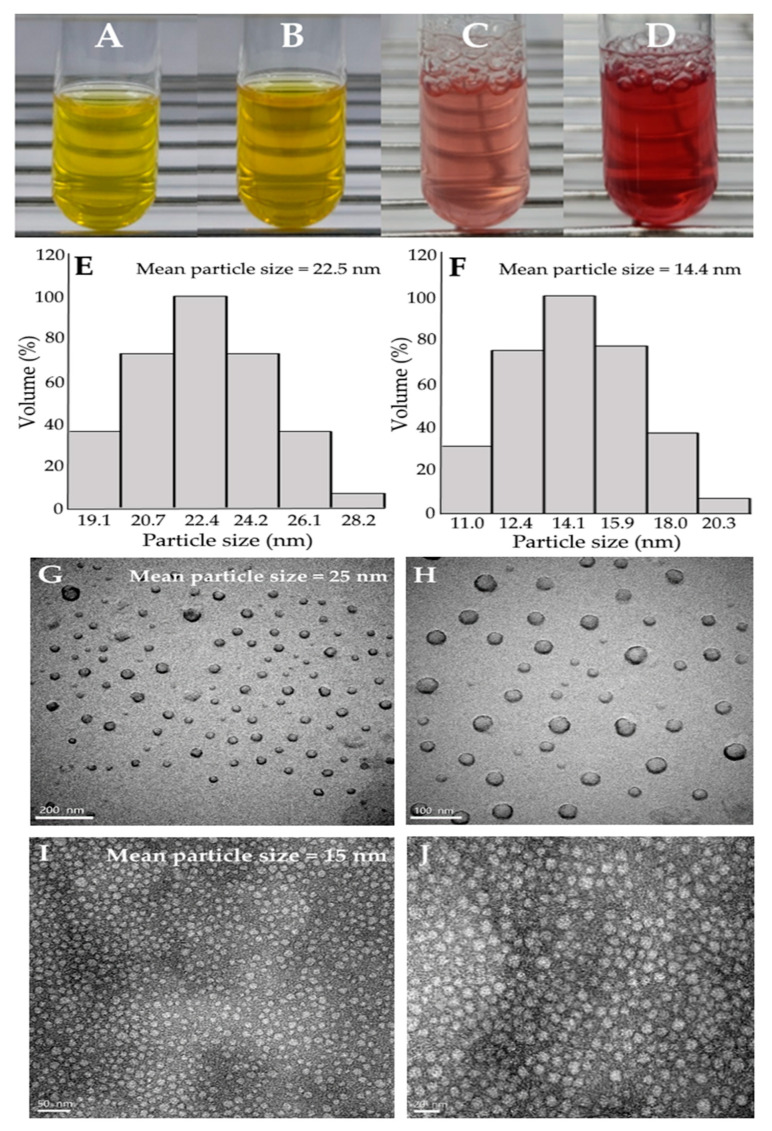
Appearance (**A**–**D**), particle size distribution (**E**,**F**) and TEM image (**G**–**J**) of xanthone nanoemulsion (**A**,**B**,**E**,**G**,**H**) and anthocyanin nanoemulsion (**C**,**D**,**F**,**I**,**J**). Xanthone nanoemulsion was prepared with a concentration of 4000 μg/mL (**A**) and 8000 μg/mL (**B**), while anthocyanin nanoemulsion was prepared with a concentration of 200 μg/mL (**C**) and 400 μg/mL (**D**).

For the preparation of nanoemulsions, highly unsaturated soybean oil was selected based on its abundance and low cost as well as its ability to protect bioactive compounds, as indicated by McClements and Jafari [32] and Hsu and Chen [33]. Moreover, various trial studies based on the calculation of HLB (hydrophilic–lipophilic balance) value were conducted to choose the appropriate surfactant/cosurfactant type and amount to stabilize the soybean oil containing xanthone or anthocyanin extract and obtain stable nanoemulsions. Figure 4 shows the appearance, particle size distribution and TEM image of xanthone and anthocyanin nanoemulsions. A transparent yellow appearance was shown for the xanthone nanoemulsion, with a concentration of 4000 μg/mL (Figure 4A) and 8000 μg/mL (Figure 4B), while a transparent red appearance was found for the anthocyanin nanoemulsion with a concentration of 200 μg/mL (Figure 4C) and 400 μg/mL (Figure 4D). The mean particle sizes of xanthone and anthocyanin nanoemulsions as determined by DLS were 22.5 and 14.4 nm, respectively (Figure 4E,F). Figure 4G,H and Figure 4I,J show the TEM images of xanthone and anthocyanin nanoemulsions, with the mean particle size being 25 and 15 nm, respectively, and round shape particles being observed for both nanoemulsions. Additionally, the PDI of xanthone and anthocyanin nanoemulsions were 0.229 and 0.153, respectively (Table 3), indicating that a narrow and uniform distribution of nanoparticles in both nanoemulsions was reached based on a report by Lakshmi and Kumar [34]. The zeta potentials of xanthone and anthocyanin nanoemulsions were −89.3 mV for the former as well as −39.4 mV (pH 2) and −61.5 mV (pH 3) for the latter (Table 3), revealing that a high stability of both nanoemulsions was attained as the zeta potential has to be controlled at >30 mV or <−30 mV to maintain the high stability of a nanosystem [35]. Compared to pH at 3, an increase in the zeta potential of the anthocyanin nanoemulsion at pH 2 is probably caused by a rise in hydrogen ions, leading to a decline of total negative charges. 

Comparatively, the mean particle sizes of xanthone and anthocyanin nanoemulsions prepared in our study were smaller than those reported by Jusril [36] and Bamba et al. [37], with the former using coconut oil, Tween 80, Span 80, and ethyl acetate extract from mangosteen peel to prepare a xanthone nanoemulsion with the mean particle size at 181 nm, and the latter using corn oil, polyglycerol polyricinoleate, and ethanol extract from blueberry pomace to prepare a water-in-oil-in-water anthocyanin nanoemulsion with the mean particle size at 400 nm. Obviously, the difference in mean particle size of the nanoemulsion can be attributed to the variation in oil, surfactant and solvent varieties as well as the extraction method. Additionally, the encapsulation efficiency of xanthone and anthocyanin nanoemulsions was 96.8 and 81.7%, respectively, both of which was higher than that reported by Bamba et al. [37], who prepared a blueberry nanoemulsion from pomace with an encapsulation efficiency of 80% being obtained. 

### 2.4. Stability of Xanthone and Anthocyanin Nanoemulsions

Table 3 also shows particle size, zeta potential and PDI changes, as affected by xanthone and anthocyanin nanoemulsions during heating at different temperatures (40–100 °C) for varied time length. A slight change in particle size, zeta potential and PDI was shown during heating at 40–100 °C for 0.5–2 h and over a 90-day storage period at 4 °C (Table 3 and Table 4), implying a high stability of the xanthone nanoemulsion prepared in our study. Similarly, for the anthocyanin nanoemulsion with pH at 2 or 3, only a slight change in particle size, zeta potential and PDI was observed, demonstrating a high stability of the anthocyanin nanoemulsion. However, compared to pH at 2, a much lower zeta potential was shown for the anthocyanin nanoemulsion with pH at 3 during heating (Table 3), revealing a higher stability of the anthocyanin nanoemulsion at pH 3. Thus, for the storage stability study, the anthocyanin nanoemulsion with pH at 3 was used. Interestingly, both particle size and PDI followed a time-dependent rise, while the zeta potential followed a time-dependent decline over a 90-day storage period at 4 °C (Table 4). Nevertheless, both PDI and zeta potential were within the acceptable range, and thus a high stability of the anthocyanin nanoemulsion was maintained during storage. The heating stability of the anthocyanin nanoemulsion prepared in our study was higher than that reported by Garcia et al. [38], who prepared anthocyanin nanoemulsion from Jaboticaba peel and the mean particle size raised from 178 to 658 nm, PDI from 0.217 to 0.350, and zeta potential from −8.6 to −2.3 mV during heating at 25–80 °C. This phenomenon indicated that, during heating, the nanoparticles may flocculate or aggregate resulting in a rise in particle size, PDI and zeta potential, and thus the stability of the nanoemulsion system was reduced substantially.

### 2.5. MTT Assay

**Figure 5:**
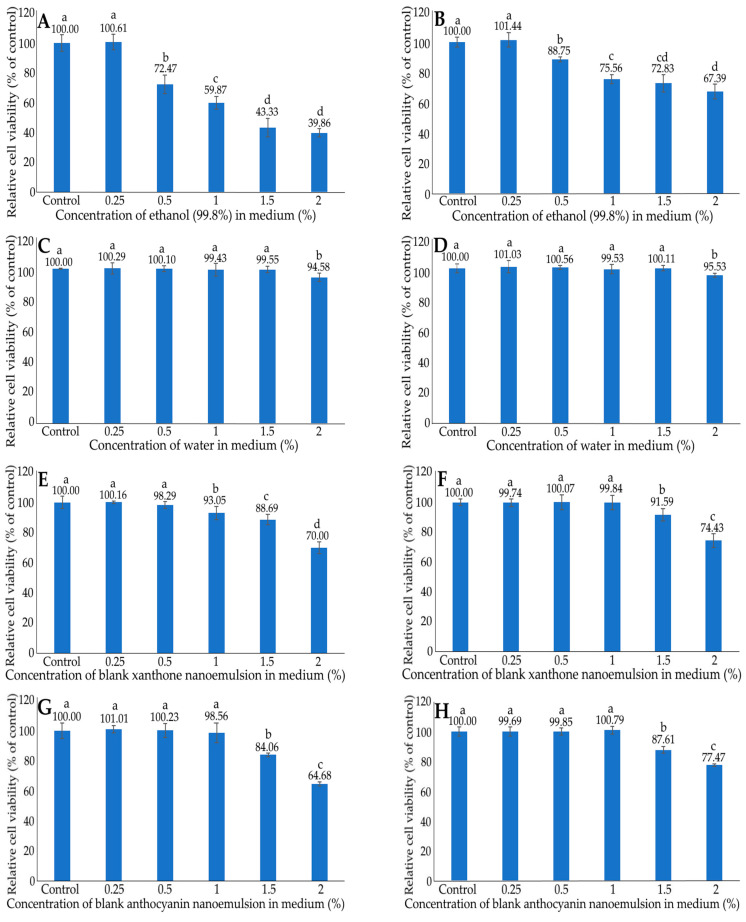
Effect of different concentrations of ethanol (**A**,**B**), deionized water (**C**,**D**), blank xanthone nanoemulsion (**E**,**F**) and blank anthocyanin nanoemulsion (**G**,**H**) on liver normal cells AML12 (**A**,**C**,**E**,**G**) and liver cancer cells HepG2 (**B**,**D**,**F**,**H**) after 48 h incubation as measured by MTT assay. Data shown are mean ± standard deviation (n = 3) and data with different small letters (a–d) are significantly different at *p* < 0.05.

Figure 5 shows the effect of different concentrations of ethanol (A,B) and deionized water (C,D) in medium on the growth of liver normal cells AML12 (A,C) and liver cancer cells HepG2 (B,D) after 48 h incubation as measured by the MTT assay. Following treatment with the ethanol concentrations at 0.25–2%, a dose-dependent reduction in cell viability was shown for both AML12 and HepG2 cells. By comparison, at the same dose, a lower cell viability was shown for AML12 cells than for HepG2 cells, indicating that ethanol possessed a higher toxicity towards AML12 cells. Thus, the ethanol concentration was controlled at 0.25% to maintain the high viability of both AML12 and HepG2 Cells to avoid the interference of ethanol for subsequent experiments. However, following treatment with the deionized water concentrations at 0.25–1.5%, no significant difference (*p* > 0.05) in the viability of either AML12 or HepG2 cells was found. However, with the deionized water concentration at 2%, the viability of AML12 and HepG2 cells decreased (*p* < 0.05) to 94.58 and 95.53%, respectively. Therefore, for subsequent experiments, the deionized water concentration was controlled at 1.5% to maintain the high viability of both AML12 and HepG2 cells.

The effects of different concentrations of blank xanthone nanoemulsion (E,F) and blank anthocyanin nanoemulsion (G,H) on the growth of liver normal cells AML12 (E,G) and liver cancer cells HepG2 (F,H) after 48 h incubation, as measured by the MTT assay, are also shown in Figure 5. A dose-dependent decline in cell viability was observed for both AML12 and HepG2 cells treated with the blank xanthone nanoemulsion at 1–2% for the former, as well as the other three blank nanoemulsions (1.5–2%) for the latter. Comparatively, at the same dose, blank xanthone nanoemulsion exhibited a higher toxicity towards AML12 cells than HepG2 cells. The same tendency was observed for the blank anthocyanin nanoemulsion. Furthermore, a significant decline (*p* < 0.05) in the viability of AML12 and HepG2 cells occurred when the blank xanthone nanoemulsion concentration was >0.5% and >1%, respectively. Thus, for subsequent experiments, the blank xanthone nanoemulsion was controlled at 0.5% to maintain the high cell viability of both AML12 and HepG2 cells. Specifically, following treatment with the blank anthocyanin concentration from 0.25 to 1%, no significant difference (*p* > 0.05) in the viability of both AML12 and HepG2 cells was observed; however, the viability of the AML12 cells significantly dropped (*p* < 0.05) to 84.06 and 64.68%, respectively, for the concentrations raised to 1.5 and 2%, as well as 87.61 and 77.47% for HepG2 cells. Therefore, the blank anthocyanin nanoemulsion was controlled at 1% for subsequent experiments to maintain the high viability of both AML12 and HepG2 cells. 

### 2.6. Inhibition of HepG2 Cells by α-Mangostin Standard, Xanthone Extract and Nanoemulsion

**Figure 6:**
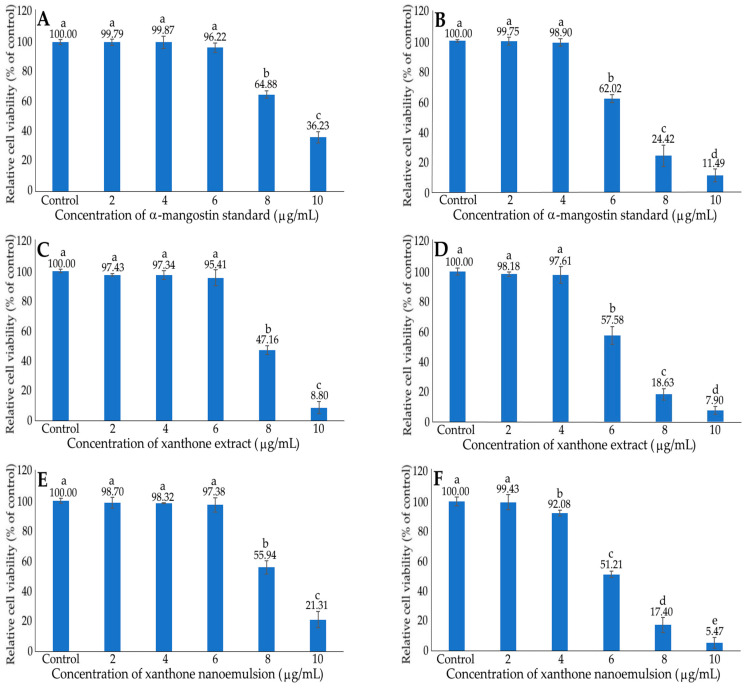
Effect of different concentrations of xanthone standard (**A**,**B**), extract (**C**,**D**) and nanoemulsion (**E**,**F**) on the growth of liver normal cells AML12 (**A**,**C**,**E**) and liver cancer cells HepG2 (**B**,**D**,**F**) after 48 h incubation, as measured by MTT assay. Data shown are mean ± standard deviation (n = 3) and data with different small letters (a–e) are significantly different at *p* < 0.05.

Figure 6 shows the effects of different concentrations of xanthone standard (A,B), extract (C,D) and nanoemulsion (E,F) on the growth of normal liver cells AML12 (A,C,E) and liver cancer cells HepG2 (B,D,F) after 48 h incubation as measured by the MTT assay. A dose-dependent reduction in cell viability was found for both AML12 and HepG2 cells when treated with the α-mangostin standard at 6–10 μg/mL. However, by comparison, at the same dose, AML12 cells showed a higher viability than HepG2 cells, implying that α-mangostin standard possessed a higher toxicity against HepG2 cells. However, for xanthone extract and nanoemulsion, no significant difference (*p* > 0.05) in cell viability was observed for AML12 cells and HepG2 cells, with the doses at 2–6 μg/mL and 2–4 μg/mL, respectively. Following a rise in the xanthone extract and nanoemulsion concentrations to 8 and 10 μg/mL, the AML12 cell viability diminished, respectively, to 47.16 and 8.80% for the xanthone extract, as well as 55.94 and 21.31% for the xanthone nanoemulsion. However, for HepG2 cells, a substantial decline in the cell viability was shown following treatment with α-mangostin standard, extract and nanoemulsion at a dose ≥6 μg/mL. Furthermore, a lower cell viability of HepG2 was found for the xanthone nanoemulsion than for the xanthone extract when compared at the same dose, revealing that the former was more toxic toward HepG2 cells. Taken together, the concentrations of α-mangostin standard, xanthone extract and xanthone nanoemulsion can be controlled at 6 μg/mL to maintain the high viability of AML12 cells and reduce the HepG2 viability pronouncedly. For the toxicity comparison, the IC_50_ of α-mangostin standard, xanthone extract and xanthone nanoemulsion in inhibiting the growth of AML12 cells were 9.05, 8.02 and 8.43 μg/mL, respectively, as well as 6.51, 6.23 and 5.78 μg/mL in inhibiting HepG2 cells. This outcome further demonstrated that the xanthone nanoemulsion was the most toxic towards HepG2 cells, followed by xanthone extract and α-mangostin standard. However, for AML12 cells, the xanthone extract exhibited the highest toxicity, followed by the xanthone nanoemulsion and α-mangostin standard. Thus, for future clinical application, the xanthone nanoemulsion should be a more ideal candidate than the xanthone extract.

In the literature reports, Cai et al. [39] explored the effect of different concentrations of α-mangostin standard on the growth of liver cancer cells including HepG2, Hep3B, MHCC-97L and Huh 7 by incubation for 24, 48 and 72 h, and a time-dependent decline in cell viability was shown with the IC_50_ being 11.76, 10.69 and 8.45 μM, respectively. Similarly, the IC_50_ was reported to be 10.94 μM following the treatment of HepG2 cells with α-mangostin standard (0–40 μM) for 72 h [40]. In an earlier study, Su et al. [41] compared the effect of 26 xanthone derivatives on the growth of cancer cells including HepG2, Lovo, KB, CNE2 and Hela, and reported that the lowest IC_50_ was shown for HepG2 cells following treatment with various xanthone standards with a dose of 4.07–200 μM. This result further illustrated that the xanthone standard possessed a more pronounced effect in inhibiting liver cancer cells than some other types of cancer cells. However, we have to point out here that most studies focus on the inhibition effect of xanthone standards on HepG2 cells, not xanthone extract or nanoemulsion.

### 2.7. Inhibition of HepG2 Cells by Anthocyanin Extract and Nanoemulsion

**Figure 7:**
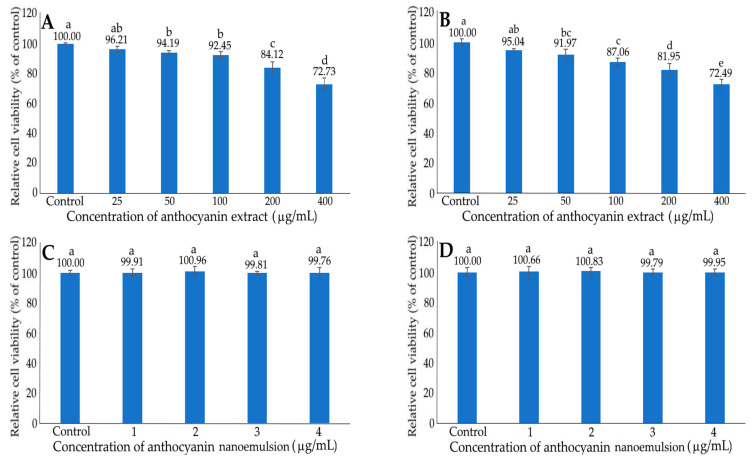
Effect of different concentrations of anthocyanin extract (**A**,**B**) and nanoemulsion (**C**,**D**) on the growth of liver normal cells AML12 (**A**,**C**) and liver cancer cells HepG2 (**B**,**D**) after 48 h incubation as measured by MTT assay. Data shown are mean ± standard deviation (n = 3) and data with different small letters (a–e) are significantly different at *p* < 0.05.

The effects of different concentrations of anthocyanin extract (A,B) and nanoemulsion (C,D) on growth in liver normal cells AML12 (A,C) and liver cancer cells HepG2 (B,D) after 48 h incubation, as measured by the MTT assay, are shown in Figure 7. Following treatment with the anthocyanin extract at 25–400 μg/mL, a dose-dependent reduction in viability was shown for both AML12 and HepG2 cells. Comparatively, a lower viability was found for HepG2 cells than for AML12 cells at the same concentration, implying that the anthocyanin extract was less toxic to AML12 cells. This outcome was further confirmed by the IC_50_ of 736.29 and 727.05 μg/mL in inhibiting AML12 and HepG2 cells, respectively. Due to the presence of a high concentration of anthocyanin (400 μg/mL) in the nanoemulsion, the highest anthocyanin concentration in the medium can only be controlled at 4 μg/mL (1%). Therefore, in this study we failed to compare the effect of a high concentration of anthocyanin nanoemulsion on the viability of both AML12 and HepG2 cells. Instead, we compared the effect of anthocyanin nanoemulsion (1–4 μg/mL) on the viability of AML12 and HepG2 cells, neither of which showed any significant difference (*p* > 0.05) after 48 h incubation. Thus, for subsequent cell culture experiments, we only studied the effect of xanthone extract and nanoemulsion on the growth of HepG2 cells. In a similar study, the cell viability of HepG2 diminished to 88, 80, 70 and 55%, respectively, following treatment with the strawberry anthocyanin extract at 100, 250, 500 and 1000 μg/mL [42]. Apparently, in our study, compared to the xanthone nanoemulsion at 4 μg/mL, the anthocyanin nanoemulsion was less effective in inhibiting the growth of HepG2 cells. 

### 2.8. Cell Cycle Analysis

**Figure 8:**
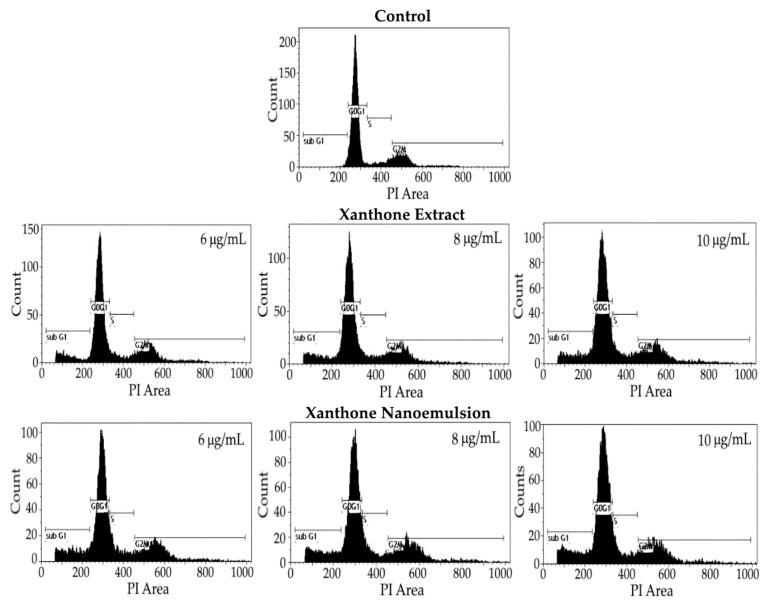
Effect of different concentrations of xanthone extract and nanoemulsion on cell cycle distribution of liver cancer cells HepG2. Control: cells were incubated in medium only.

Figure 8 and Table 5 show the effect of different concentrations of xanthone extract and nanoemulsion on the cell cycle distribution of liver cancer cells HepG2. The proportion of the sub-G1 phase was only 1.10% for the control treatment; however, a large increase by 9.56, 10.51 and 10.40% was shown, respectively, for the xanthone extract at 6, 8 and 10 μg/mL, as well as 13.02, 14.63 and 15.43% for the xanthone nanoemulsion at the same doses. This outcome indicated that both xanthone extract and nanoemulsion were effective in inducing the apoptosis of HepG2 cells, with the latter being more efficient. For the proportion of G0/G1 phase, a significant decline (*p* < 0.05) by 10.92, 11.41 and 13.58%, respectively, was found following treatment with the xanthone extract at 6, 8 and 10 μg/mL, as well as 17.17, 19.17 and 19.84% when treated with the xanthone nanoemulsion at the same doses. By comparison, xanthone nanoemulsion was more effective than xanthone extract in decreasing the proportion of the G0/G1 phase, probably caused by the enhanced permeation and retention (EPR) effect for easier penetration into cells [33]. Similar to the sub-G1 phase, a significant rise (*p* < 0.05) in the S phase proportion by 1.48, 1.73 and 3.08%, respectively, was found for the xanthone extract at 6, 8 and 10 μg/mL, as well as 2.70, 3.11 and 3.21% for the xanthone nanoemulsion at the same doses, when compared to the control treatment. However, both xanthone extract and nanoemulsion showed a similar effect in increasing the S phase proportion. Interestingly, compared to the control, following treatment with xanthone extract or nanoemulsion at 6, 8, and 10 μg/mL there was no significant difference (*p* > 0.05) in the proportion of the G2/M phase. Collectively, a dose-dependent rise in the proportion of the sub-G1 phase and a dose-dependent decline in the proportion of the G0/G1 phase occurred for both xanthone extract and nanoemulsion treatments. Thus, it may be inferred that the cell cycle of HepG2 cells may be arrested at the S phase. As mentioned above, most studies are focused on the effect of xanthone standards, not xanthone extract or nanoemulsion, on inhibition of cancer cell growth. For instance, a dose-dependent decline in the proportion of the G1 phase was shown, while a dose-dependent rise in the proportion of the S phase was found following treatment of HepG2 cells with the xanthone standard at 2, 4 and 8 μM for 48 h. Similarly, for some other types of cancer cells, after the treatment of colon cancer cells COLO 205 with α-mangostin at 10, 20 and 30 μg/mL the proportion of the sub-G1 phase showed the most prominent increment, while that of G0/G1, S and G2/M phases were reduced to a minimum [43]. However, no definite conclusion was made regarding the arrest of COLO 205 cells at a certain phase. Nevertheless, the ovarian cancer cells SKOV-3 were postulated to be arrested at S and G2/M phases following treatment with α-mangostin standard, as both proportions of S and G2/M phases showed a dose-dependent rise. Similarly, the medulloblastoma cells Daoy were postulated to be arrested at the G2/M phase after treatment with α-mangostin standard at 10, 15 and 20 μM, as evidenced by a dose-dependent increase in the proportion of the G2/M phase after 24 h incubation. Collectively, the arrest of liver cancer cells and some other types of cancer cells at a certain phase as affected by xanthone can be dependent upon xanthone variety, xanthone standard or extract, xanthone concentration, incubation time and the method of xanthone extract or nanoemulsion preparation.

### 2.9. Analysis of Apoptosis of Liver Cancer Cells

**Figure 9:**
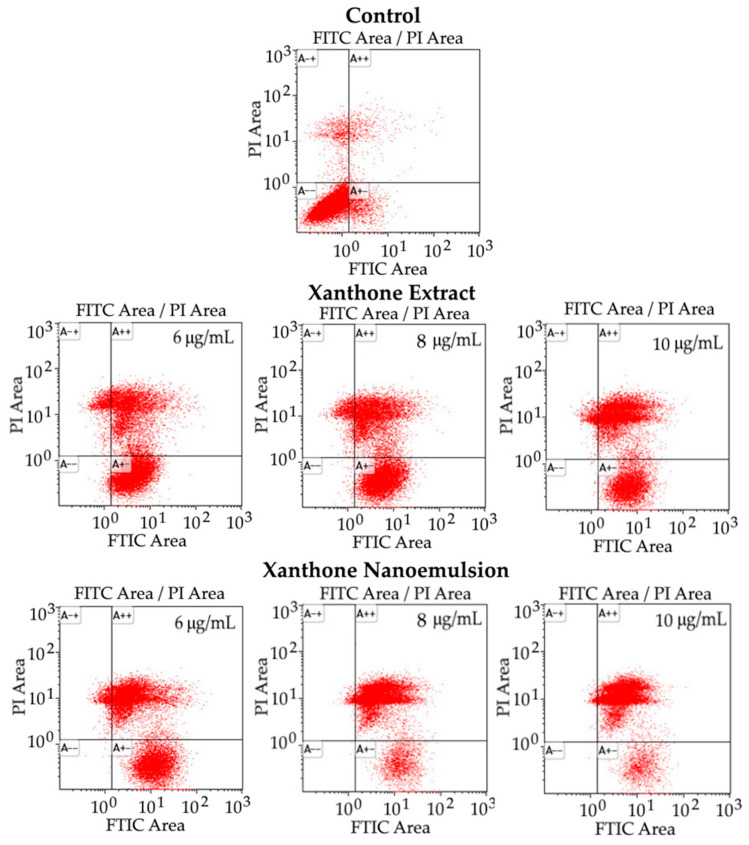
Apoptosis of liver cancer cells HepG2 treated with different concentrations of xanthone extract and xanthone nanoemulsion. Control: cells were incubated in medium only. A− +, necrosis cells; A+ +, late apoptosis cells; A− −, viable cells; A+ −, early apoptosis cells.

The apoptosis of liver cancer cell HepG2 as affected by different concentrations of xanthone extract and nanoemulsion is shown in Figure 9 and Table 6. Compared to the control treatment, a large decline in the proportion of viable cells was shown for both xanthone extract and nanoemulsion at 6, 8, and 10 μg/mL with a dose-dependent response. By comparison, the xanthone nanoemulsion was more effective than xanthone extract in decreasing the proportion of viable cells at the same dose. Conversely, all the proportions of early apoptosis, late apoptosis and necrosis cells rose substantially, especially for late apoptosis cells, following treatment with both xanthone extract and nanoemulsion at 2, 4 and 6 μg/mL. Specifically, a dose-dependent drop in the proportion of early apoptosis cells was found for both xanthone extract and nanoemulsion, with the proportion of the former being higher at the same dose. However, for the proportion of late apoptosis cells, a dose-dependent rise occurred for both xanthone extract and nanoemulsion, with the former proportion being lower at the same dose. This finding demonstrated that most HepG2 cells could undergo late apoptosis when treated with the xanthone nanoemulsion at 6, 8 and 10 μg/mL, while with the xanthone extract most HepG2 cells underwent early apoptosis at 6 and 8 μg/mL as well as late apoptosis at 10 μg/mL. Interestingly, the proportion of necrosis cells showed inconsistent change for the xanthone extract, but for the xanthone nanoemulsion a dose-dependent decline was found, with the former proportion being higher at 8 and 10 μg/mL. Comparatively, the xanthone extract was more effective than the xanthone nanoemulsion in inducing HepG2 cells to undergo necrosis. Nevertheless, a large proportion of HepG2 cells underwent late apoptosis following treatment with the xanthone nanoemulsion, probably caused by the EPR effect, as mentioned above. The result observed in this study is similar to a report by Jin et al. [44], who prepared the xanthone extract from the bark of *Garcinia xanthochymus* with 95% ethanol; a dose-dependent increment in the proportion of apoptotic cells was shown, with the proportion of late apoptosis cells being higher than early apoptosis cells. Similar findings were reported by Abu Bakar et al. [45] and Zhang et al. [40], with the former using xanthone extract prepared from *Garcinia dulcis* fruit for the treatment of HepG2 cells for 72 h, and the latter using α-mangostin standard for the treatment of HepG2 and SK-Hep-1 for 24 h; a large rise in the proportion of late apoptosis cells was found when compared to the proportions of early apoptosis cells and necrosis cells.

### 2.10. Activities of Caspase-3, Caspase-8 and Caspase-9

**Figure 10:**
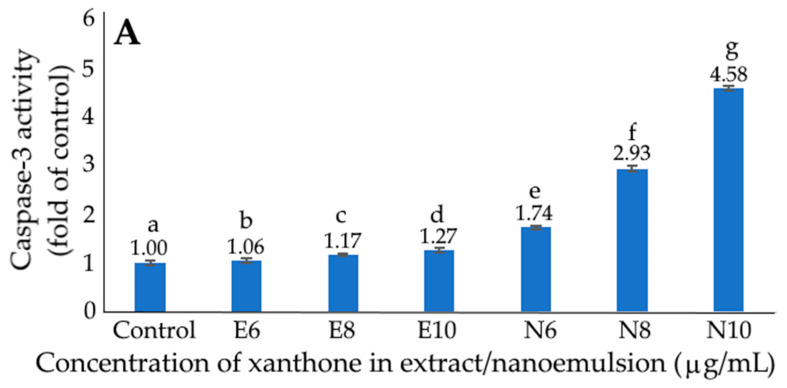

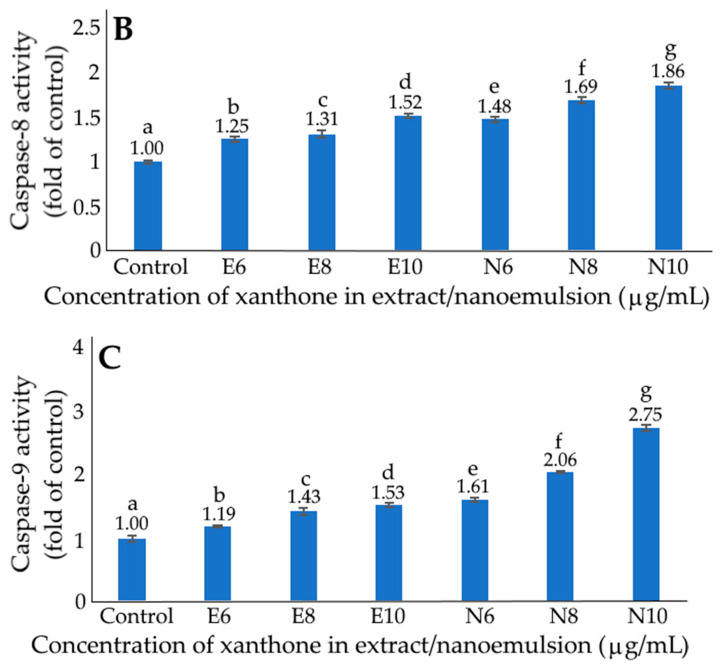
Effect of xanthone extract and nanoemulsion on caspase-3 (**A**), caspase-8 (**B**) and caspase-9 (**C**) activities of liver cancer cells HepG2. E6, E8 and E10 represent xanthone extract concentrations at 6, 8 and 10 μg/mL, respectively, while N6, N8 and N10 represent xanthone nanoemulsion concentrations at 6, 8 and 10 μg/mL. Data shown are mean ± standard deviation (n = 3) and data with different small letters (a–g) are significantly different at *p* < 0.05.

Figure 10 shows the effects of xanthone extract and nanoemulsion on caspase-3 (A), caspase-8 (B) and caspase-9 (C) activities of liver cancer cells HepG2. Compared to the control treatment, both xanthone extract and nanoemulsion showed a significantly higher caspase-3 activity (*p* < 0.05) at 6, 8 and 10 μg/mL. Additionally, a dose-dependent increment in caspase-3 activity was found for both xanthone extract and nanoemulsion treatments, with the latter showing a higher activity at the same dose. The same tendency was observed for both caspase-8 and caspase-9 activities, as evident by a dose-dependent rise for both xanthone extract and nanoemulsion, as well as a higher activity for xanthone nanoemulsion when compared at the same dose. Collectively, during apoptosis, caspase-8 can be activated through the death receptor pathway (external route) while caspase-9 can be activated through the mitochondrial pathway (internal route) for the subsequent activation of caspase-3 and the execution of apoptosis.

In several similar studies, the activities of caspase-3, caspase-8 and caspase-9 also followed a dose-dependent increase after the treatment of HepG2 cells with α-mangostin standard at 5, 7 and 14 μM for 24 h [46]. Likewise, Jin et al. [44] used 95% ethanol to extract xanthone from the bark of *Garcinia xanthochymus* and observed a dose-dependent rise in the activities of caspase-3, caspase-8 and caspase-9 following the treatment of HepG2 cells with the xanthone extract at 6.25, 12.5 and 25 μmol/L for 12 h. It was postulated that, with the xanthone extract treatment, caspase-8 was activated first for the subsequent signal transduction to mitochondria for the regulation of the BcL-2 family, which in turn activated caspase-9 in cytoplasm, leading to caspase-3 activation for apoptosis execution.

Taken together, a total of seven xanthones and two anthocyanins isolated from mangosteen peel were quantified by UPLC-MS/MS, followed by preparation of nanoemulsions and the evaluation of their inhibition effects on liver cancer cells HepG2. The xanthone nanoemulsion was shown to be more effective in inhibiting HepG2 cells than xanthone extract, while anthocyanin nanoemulsion at the same concentration failed to inhibit the growth of HepG2 cells. The inhibition mechanism of HepG2 cells by xanthone extract and nanoemulsion revealed cell cycle arrest at S phase, accompanied by a higher proportion of late apoptosis cells and an increase in caspase-3, caspase-8 and caspase-9 activities, resulting in cell apoptosis. Future studies can be directed towards evaluating the anti-tumor effect in vivo, as well as modifying the nanoemulsion system by incorporating a ligand such as hyaluronic acid for active targeting CD44 receptors overexpressed in liver cancer cells, as pointed out by Rosso et al. [22]. Additionally, based on a report by Vecchione et al. [23], the nanoemulsion system can be modified by incorporating magnetic nanoparticles such as iron oxide or cobalt ferrite oxide for exerting both therapeutic and imaging efficiency. On the other hand, nanoemulsions with high loading of anthocyanin can be prepared for evaluating the inhibitory effects on the growth of liver cancer cells HepG2. This is because anthocyanin-encapsulated nanoemulsion can reduce the potential side effects of cardiotoxicity caused by some common chemotherapeutic drugs, including doxorubicin [47]. It is worth pointing out that anthocyanins have been reported to exhibit cardioprotective effects by exerting antioxidant activity through reducing oxidative stress in cardiac cells, and anti-inflammatory activity through preventing the generation of proinflammatory cytokines, as well as protecting the heart from ischemia/reperfusion-induced injury through the activation of signal transduction pathways, and sustaining mitochondrial function through the reduction of cytosolic cytochrome C, as well as the maintenance of electron transfer between NADH dehydrogenase and cytochrome C [47,48,49].

## 3. Materials and Methods

### 3.1. Materials

A total of 1 kg of mangosteen peel was purchased from Wu-Chia-Mu Agricultural Product Co., located in Kaohsiung, Taiwan, vacuum packed and transported to our laboratory under freezing condition. Following thawing, washing, cutting into pieces and freeze-drying, mangosteen peels were ground into powder.

Xanthone standards including α-mangostin and γ-mangostin were obtained from Sigma-Aldrich Co. (St. Louis, MO, USA), while β-mangostin, garcinone C, garcinone D, gartanin and 8-desoxygartanin were from Chen-Du Pu-Ruei-Fa Scientific Co. (Sichuan, China). Internal standard xanthone was also procured from Sigma-Aldrich Co. Anthocyanin standards including cyanidin-3-sophoroside chloride and cyanidin-3-glucoside chloride were from Tokiwa Phytochemical Co. (Chiba, Japan), while the internal standard pelargonidin-3-glucoside was from Extrasynthese Co. (Genay, France).

The HPLC-grade solvents, including methanol and acetonitrile, were from Riedel-de Haen Co. (Muskegon, MI, USA), while both formic acid and hydrochloric acid were from Sigma-Aldrich Co. Deionized water was made using a Milli-Q water purification system from Millipore Co. (Bedford, MA, USA). Soybean oil was from Chuan-Liang Welfare Center (Taipei, Taiwan), while CITREM was from Chen-Ding Co. (Taipei, Taiwan). Both Tween 80 and glycerol were from Yu-Pa Co. (Taipei, Taiwan), while lecithin was from Chen-Fang Co. (Taipei, Taiwan). Chemicals including PEG 400, citric acid, potassium dihydrogen phosphate and sodium hydrogen phosphate were from Sigma-Aldrich Co.

Human liver cancer cell (HepG2) and mice liver cell (AML 12) were purchased from the Bioresource Collection and Research Center (Hsinchu, Taiwan). Minimum essential medium (MEM), sodium bicarbonate solution, non-essential amino acid (2 mM L-glutamine, 100 × solution), sodium pyruvate solution, Dulbecco’s modified Eagle’s medium and Ham’s F12 medium (1:1), fetal bovine serum (FBS), phosphate-buffered saline (PBS), Hank’s balance salt solution (HBSS) and 0.25% Trypsin-EDTA were from Hyclone Co. (Logan, UT, USA). Insulin, transferrin, selenium and dexamethasone were from Sigma-Aldrich Co.

Dimethyl sulfoxide (DMSO), 0.4% trypan blue, thiazolyl blue tetrazolium bromide (MTT), bovine serum albumin (BSA), and propidium iodide (PI) were from Sigma-Aldrich Co. The Bradford reagent for protein assay was from Bio-Rad Co. (Hercules, CA, USA). The fluorometric assay kits, including caspase-3, caspase-8 and caspase-9, were from Bio Vision Co. (Milpitas, CA, USA), while the Annexin V ifluor 488 + PI apoptosis detection reagent was from Croyez Bioscience Co. (Taipei, Taiwan).

### 3.2. Sample Pretreatment

A total amount of 923 g of mangosteen was obtained after thawing and washing, followed by separating mangosteen peel from pulp with the hands to obtain a total amount of 645 g of peel. Then, mangosteen peels were cut into pieces (1 cm each side), freeze dried, ground into powder (227 g), packed in sealed bags and stored in a freezer (−30 °C) until use.

### 3.3. Extraction of Xanthones and Anthocyanins

For the extraction of anthocyanins, a 1 g sample of mangosteen peel powder was poured into a 50 mL centrifuge tube, then 40 mL of methanol or ethanol was added for comparison of extraction efficiency. After shaking at 220 rpm for 1 h, the solution was centrifuged at 4000 rpm (4 °C) for 20 min and the supernatant was collected in a round-bottom flask. Then, 20 mL of methanol or ethanol was added to the residue for extraction, centrifuging was carried out and the supernatant was collected. Both supernatants were combined and evaporated to dryness, followed by the addition of 10 mL of deionized water, ultrasonic vibration for 10 min, centrifuging at 4000 rpm (4 °C) for 20 min, and then the supernatant (red) was collected as the anthocyanin extract. The yellow residue was used for xanthone extraction.

For xanthone extraction, 40 mL of deionized water was added to the yellow residue and the solution was shaken in an ultrasonicator for 10 min, followed by centrifuging at 4000 rpm (25 °C) for 40 min, and the supernatant was then removed. Then, 20 mL of deionized water was added to the residue and the solution was shaken in an ultrasonicator for 10 min, followed by centrifuging at 4000 rpm (25 °C) for 40 min, and the supernatant was removed. Then, the residue was freeze-dried to obtain the xanthone extract by dissolving in 10 mL of ethanol, filtered through a 0.22-μm PVDF membrane filter and stored at −20 °C until use.

### 3.4. Separation and Identification of Xanthones and Anthocyanins by UPLC-MS/MS

The separation of various anthocyanins in mangosteen peels was carried out using a method similar to that described by Li et al. [50]. A Gemini C18 110A column (250 × 4.6 mm ID, particle size 5 μm) from Phenomenex Co. (Torrance, CA, USA) and a gradient mobile phase of aqueous solution containing 2% formic acid (A) and acetonitrile containing 2% formic acid (B) with flow rate at 0.5 mL/min and column temperature at 35 °C was used: 85% A and 15% B initially, increased to 80% A and 20% B in 3 min, and maintained until 12 min.

For the separation of various xanthones in mangosteen peels, an HPLC method based on Walker [6] was used. A Kinetex C18 100A column (100 × 4.6 mm ID, particle size 2.6 μm) from Phenomenex Co. (Torrance, CA, USA) and a gradient mobile phase of aqueous solution containing 0.1% formic acid (A) and methanol (B) with a flow rate at 0.5 mL/min and column temperature at 30 °C was used: 20% A and 80% B initially, raised to 18% A and 82% B in 1 min, 17% A and 83% B in 2 min, 15.5% A and 84.5% B in 3 min, maintained till 9 min, raised to 7% A and 93% B in 10 min, 3% A and 97% B in 11 min, and 100% B in 12 min, maintained until 15 min.

Both anthocyanins and xanthones in mangosteen peels were identified by UPLC-MS/MS (Accela 600 series UPLC coupled with LTQ Orbitrap XL) from Thermo Fisher Scientific Co. (San Jose, CA, USA) using the following conditions: the ion source was electrospray ionization (ESI) with selected reaction monitoring (SRM) mode; the spray voltage was 3500 V for positive ion mode and 2800 V for negative ion mode; the flow rates of sheath gas, auxiliary gas and sweep gas were 45, 20 and 2 arbitrary units, respectively; the temperatures of ion transfer tube and vaporizer were 350 °C and 300 °C, respectively. Of the various anthocyanins and xanthones, only the positive ion mode was selected for the internal standard xanthone, while the negative ion mode was selected for the other compounds. Identification was carried out by comparing retention time and mass to charge ratios (precursor ions and product ions) of unknown peaks with those of xanthone and anthocyanin standards.

### 3.5. Method Validation

For recovery determination, two levels of various standards including α-mangostin (5000 and 50,000 μg), γ-mangostin (4000 and 40,000 μg), garcinone C and garcinone D (200 and 2000 μg each), 8-desoxygartanin, gartanin and β-mangostin (400 and 4000 μg each), cyanidin 3-sophoroside (500 and 5000 μg), and cyanidin 3-glucoside (200 and 2000 μg) were added to 1 g of mangosteen peel powder. Following extraction and quantitation by UPLC-MS/MS, the recoveries of individual xanthone and anthocyanin in mangosteen peels were obtained based on the relative ratio of the amount of the standards after UPLC-MS/MS to the amount of the standards added before UPLC-MS/MS.

For precision study, both intra-day and inter-day variabilities were determined by analyzing various xanthones and anthocyanins in mangosteen peels in the morning, afternoon and evening in triplicate on the same day for a total of 9 analyses for the former, while for the latter the various xanthones and anthocyanins were determined on the 1st, 2nd and 3rd day in triplicate for a total of 9 analyses. Then, the standard deviation (SD) and relative standard deviation (RSD%) were calculated.

For LOD determination, a total of 13 concentrations including 0.0001, 0.0005, 0.001, 0.002, 0.004, 0.006, 0.008, 0.01, 0.02, 0.03, 0.04, 0.05 and 0.1 μg/mL of each xanthone standard were prepared, while 9 concentrations including 0.01, 0.02, 0.03, 0.04, 0.05, 0.1, 0.2, 0.5 and 1.0 μg/mL were prepared for each anthocyanin standard. Following UPLC-MS/MS analysis in triplicate, the standard calibration curves were obtained by plotting concentration against mean peak area, with the linear regression equations, slope (S) and standard deviation (δ) of the response values being used for the calculation of LOD and LOQ using the following formula:

$$\mathrm{LOD}=\frac{3.3\times \delta }{S}$$

LOQ=3×LOD

### 3.6. Quantitation

An internal standard xanthone was used for the quantitation of xanthones, and pelargonidin-3-glucoside for the quantitation of anthocyanins in mangosteen peels. A total of 6 concentrations (5, 10, 20, 30, 40 and 50 μg/mL) of cyanidin-3-sophoroside dissolved in methanol containing 0.1% hydrochloric acid were prepared, while a total of 6 concentrations (0.05, 0.1, 0.2, 0.5, 1.0 and 5.0 μg/mL) of cyanidin-3-glucoside dissolved in methanol containing 0.1% hydrochloric acid were prepared. Then, pelargonidin-3-glucoside was added to each standard concentration with the internal standard concentration fixed at 10 μg/mL. Likewise, a total of 6 concentrations (0.2, 0.5, 1.0, 5.0, 20 and 50 μg/mL) of xanthone standards dissolved in ethanol were prepared, followed by adding xanthone to each standard concentration with the internal standard concentration maintained at 10 μg/mL. Following the injection of each xanthone or anthocyanin concentration into UPLC-MS/MS in triplicate, the standard curves of each xanthone and anthocyanin were obtained by plotting the concentration ratio against area ratio, with the linear regression equations and coefficient of determination (R^2^) being obtained.

For quantitation, a concentration (10 μg/mL) of the internal standard xanthone was added to the xanthone extract, and pelargonidin-3-glucoside (10 μg/mL) to the anthocyanin extract. Following UPLC-MS/MS analysis, both xanthones and anthocyanins in mangosteen peels were quantified using the following formula:

$$\mathrm{Xanthones}/\mathrm{Anthocyanins}\;(\mu g/g)=\left(\frac{A_{s}}{A_{i}}-b\right)\times \frac{1}{a}\times C_{i}\times V\times \mathrm{DF}\times \frac{1}{\mathrm{recovery}}\times W_{s}$$

where A_s_: peak area of individual xanthone/anthocyanin in sample; A_i_: peak area of internal standard; b: intercept of the linear regression equation; a: slope of the linear regression equation; Ci: concentration of the internal standard; V: quantitation volume of the extract; DF: dilution factor; and W_s_: weight of the sample (g).

### 3.7. Preparation of Xanthone and Anthocyanin Nanoemulsions

For the preparation of the xanthone nanoemulsion, 1.167 mL of the xanthone extract containing xanthone at 68,572 μg/mL was collected, poured into a test tube and evaporated to dryness under nitrogen. Then, 0.1 g (1%) of soybean oil was added to dissolve the residue by stirring, followed by adding 0.3 g (3%) of Tween 80 and 0.075 g (0.75%) of CITREM, stirring again, and adding 9.525 g (95.25%) of deionized water. Following stirring thoroughly, this solution was transferred to a centrifuge tube for probe-sonicating for 30 s and cooling for 30 s, with this procedure being repeated for a total of 2 min. Then, a 10-mL xanthone nanoemulsion containing xanthone at 8000 μg/mL was prepared.

Similarly, for preparation of the anthocyanin nanoemulsion, 1.368 mL of the anthocyanin extract containing anthocyanin at 2924 μg/mL was collected, followed by adding 0.075 g (0.75%) of deionized water to dissolve the residue, 0.055 g (0.55%) of ethanol, 0.1 g (1%) of soybean oil, 0.2 g (2%) of PEG400, 0.055 g (0.55%) of lecithin, 0.6 g (6%) of Tween 80, 0.065 g (0.65%) of glycerol and 8.85 g (88.5%) of deionized water. After mixing thoroughly, this solution was subjected to ultrasonication for 15 min and then a 10-mL anthocyanin nanoemulsion containing anthocyanin at 400 μg/mL was prepared.

### 3.8. Determination of Nanoemulsion Characteristics

A portion (20 μL) of xanthone and anthocyanin nanoemulsions was collected separately and mixed with 980 μL of potassium dihydrogen phosphate (25 mM, pH 5.3–5.5), after which this mixture was transferred to a colorimetric tube for determination of mean particle size and polydispersity index (PDI) by a dynamic light scattering (DLS) instrument. Similarly, a portion (20 μL) of xanthone and anthocyanin nanoemulsions was collected separately and mixed with 980 μL of deionized water for the determination of zeta potential by an analyzer. For the determination of the TEM image, a 10-μL sample of xanthone nanoemulsion and 100-μL sample of anthocyanin nanoemulsion were collected separately, followed by dilution with deionized water by 200-fold for the former and 10-fold for the latter. Then, a 20-μL sample from both nanoemulsions was collected and dropped onto a carbon-coated copper grid for settling, followed by removing the excess sample with a filter paper, dropping 20 μL of phosphotungstic acid (2%), removing the excess sample again with a filter paper, and drying in an oven overnight for determination by a transmission electron microscope under 120 kV by enlarging 3 × 10^5^ times.

For the determination of encapsulation efficiency, a 100 μL sample of xanthone and anthocyanin nanoemulsions was collected separately and mixed with 900 μL of potassium dihydrogen phosphate (25 mM, pH 5.3–5.5), followed by transferring to a centrifuge tube containing dialysis membrane (10 kDa) for centrifuge at 12,000 rpm (25 °C) for 20 min. Unencapsulated xanthone and anthocyanin can pass through the dialysis membrane, and the lower layer was collected for the quantitation of xanthone and anthocyanin and calculation of encapsulation efficiency by using the following formula:

$$\mathrm{Encapsulation}\;\mathrm{efficiency}\;(\%)=\frac{\mathrm{total}\;\mathrm{amount}-\mathrm{free}\;\mathrm{amount}\;}{\mathrm{total}\;\mathrm{amount}}$$

### 3.9. Stability of Xanthone and Anthocyanin Nanoemulsions

A 300-μL sample of sample nanoemulsion was placed in a water bath and heated at 40, 60, 80 and 100 °C for 30, 60, 90 and 120 min, after which both PDI and zeta potential were determined. Similarly, the heating stability of anthocyanin nanoemulsion was determined using the same approach, with the exception that the pH of anthocyanin nanoemulsion was controlled at 2 or 3 through the addition of citric acid.

For storage stability, 3 mL of xanthone nanoemulsion was collected and stored at 4 °C for 90 days, during which both PDI and zeta potential were determined every 15 days. Similarly, the storage stability of anthocyanin nanoemulsion was determined with the exception that the pH of anthocyanin nanoemulsion was controlled at 3 through the addition of citric acid.

### 3.10. Protection of Xanthone and Anthocyanin Extracts/Nanoemulsions

Both xanthone and anthocyanin extracts/nanoemulsions were protected from possible degradation in light by performing all the experiments under dimmed light and by using brown-colored bottles/tubes. They were also protected from high-temperature degradation by evaporating solvents in a vacuum evaporator or by passing nitrogen and centrifuging at 4 °C. Moreover, all the extracts were stored at −20 °C, while nanoemulsions were stored at 4 °C.

### 3.11. Cell Culture Experiment

Human liver cancer cells HepG2 were cultured in 90% MEM and 10% FBS, while mice liver cells AML12 were cultured in 90% of DMEM and F12 (1:1, *v*/*v*) and 10% FBS. Then, cryogenic tubes were collected from liquid nitrogen and thawed at 37 °C, followed by placing in a 10 cm plate, adding 9 mL of medium, and incubating in an incubator (37 °C) with 100% relative humidity and 5% carbon dioxide. After reaching 80% confluency, the subculture experiment was started by sucking medium and adding 2 mL of PBS. Then, the PBS was removed, followed by the addition of 2 mL of trypsin-EDTA, placing in an incubator for 3–5 min, adding 2 mL medium to terminate the trypsin-EDTA reaction, and collecting the cytosol for centrifuging at 1500 rpm for 5 min (25 °C). The supernatant was then removed and 1 mL medium added for thrashing cells evenly for subsequent seeding in a new plate.

### 3.12. MTT Experiment

Initially, a 25-mg MTT powder was dissolved in 5 mL PBS and filtered through a 0.22-μm membrane filter (PVDF) for storage at −20 °C. Then, a 40-mL medium containing 1.5 × 10^4^ cells/mL was prepared and transferred to a 96-well plate with 200 μL each well for 24 h. Following cell attachment, the medium was removed and washed with PBS, followed by the addition of different concentrations of xanthone extracts or nanoemulsions (2, 4, 6, 8, 10 μg/mL) for both and anthocyanin extracts (25, 50, 100, 200 and 400 μg/mL) or nanoemulsions (1, 2, 3 and 4 μg/mL) for triplicate experiments. After incubation for 48 h, the medium was removed and washed with PBS, followed by the addition of the MTT solution to the 96-well plate with 200 μL each, incubation in the dark for 1 h, removal of the MTT solution, and addition of DMSO (100 μL) to dissolve the purple crystals for the absorbance measurement at 570 nm. The cell viability was then calculated using a formula as described by Yang et al. [51].

### 3.13. Cell Cycle Experiment

Human liver cancer cells HepG2 (1 × 10^6^) were cultured in a 6-well plate and incubated for 24 h for cell attachment, after which the medium was sucked and added with three concentrations (6, 8 and 10 μg/mL) of xanthone extracts or nanoemulsions for triplicate experiments. After incubation for 48 h, the medium was collected in a centrifuge tube and trypsin-EDTA (0.5 mL) added for incubating (37 °C) for 5–10 min. Following cell floating, the reaction was terminated with medium (0.5 mL), followed by the collection of cytosols in a centrifuge tube for centrifuging at 1500 rpm (25 °C) for 3 min, removal of the supernatant, addition of PBS (300 μL), addition of 720 μL of ethanol slowly, and storage at −20 °C overnight to fix cells. Following thawing, the cytosols were centrifuged again at 1500 rpm (4 °C) for 5 min, followed by removal of the supernatant, addition of PBS (0.5 mL), and centrifuging again using the same condition. Then, PBS (0.8 mL) was added, followed by addition of RNase A and propidium iodide for reaction in the dark (37 °C) for 30 min. for the determination of the proportions of Sub-G1, G0/G1, S and G2/M phases using Kuluza analysis software (version 3.1, Beckman Coulter Inc., Taipei, Taiwan).

### 3.14. FITC-Annexin V/PI Experiment

Human liver cancer cells HepG2 (1 × 10^6^) were cultured in a 6-well plate and incubated for 24 h for cell attachment, after which the medium was removed and added with three concentrations (6, 8 and 10 μg/mL) of xanthone extracts or nanoemulsions for triplicate experiments. Following 48 h incubation, the medium was collected in a centrifuge tube and trypsin-EDTA (0.5 mL) added for incubating (37 °C) for 5–10 min. After cell floating, the reaction was terminated with medium (0.5 mL), followed by the collection of cytosols in a centrifuge tube for centrifuging at 1500 rpm (25 °C) for 3 min, removal of the supernatant and adding PBS (300 μL). Then, 0.1 mL of 1X binding buffer was added for cell dispersion, followed by addition of 5 μL of FITC-Annexin V and 5 μL of propidium iodide for reaction in the dark (25 °C) for 15 min, and addition of 0.4 mL of 1X binding buffer for analysis of the proportions of viable cells (Annexin V−/PI−), early-apoptosis cells (Annexin V+/PI−), late-apoptosis cells (Annexin V+/PI+) and necrosis cells (Annexin V−/PI+) by a flow cytometer coupled with Kuluza analysis software.

### 3.15. Determination of Caspase-3, Caspase-8 and Caspase-9 Activities

Human liver cancer cells HepG2 (5 × 10^5^) were seeded in a 6-well plate. Following the same approach of incubation, collection and washing cells, the cell lysis buffer (50 μL) was added and transferred to an ultrasonicator for shaking for 30 min (4 °C) for protein extraction. Following centrifuging at 12,000 rpm (4 °C) for 30 min, the supernatant was collected and stored at −80 °C for subsequent experiments.

A total of 8 concentrations (6.25, 12.5, 25, 50, 75, 100, 125 and 150 μg/mL) of bovine serum albumin (BSA) standards were prepared, after which 100 μL from each concentration was collected and added to 400 μL of the diluted Bradford staining agent for reaction for 5 min. Then, each 100 μL solution was transferred to a 96-well plate for the absorbance measurement at 595 nm by an ELISA reader. The standard calibration curves of BSA were prepared by plotting concentration against absorbance for protein quantitation in cell extract.

Next, the activities of caspase-3, caspase-8 and caspase-9 were determined using three different fluorescence assay kits. In brief, a 50-μL cell extract containing 50 μg of protein was collected and mixed with 50 μL of 2X reaction buffer containing 10 mM DTT, followed by adding 5 μL of three different substrates including DEVD-AFC (caspase-3), IETD-AFC (caspase-8) and LEHD (caspase-9) for reaction at 37 °C for 1 h, and transferring to a 96-well plate for the absorbance measurement at 400 nm (excited wavelength) and 505 nm (emission wavelength) by an ELISA reader.

### 3.16. Statistical Analysis

All the data were subjected to statistical analysis by a statistical analysis system 9.4 software [52] for ANOVA analysis and Duncan’s multiple range test for significance in mean comparison (*p* < 0.05).

## 4. Conclusions

In conclusion, a Kinetex C18 column coupled with UPLC-ESI-MS/MS and a gradient mobile phase of 0.1% formic acid solution (A) and methanol (B) can be used to separate seven xanthones within 13 min, including garcinone C, garcinone D, γ-mangostin, 8-desoxygartanin, gartanin, α-mangostin and β-mangostin, while a Gemini C18 column coupled with UPLC-MS/MS and a gradient mobile phase of 2% formic acid solution (A) and 2% formic acid in acetonitrile (B) can be used to separate two anthocyanins, including cyanidin-3-sophoroside and cyanidin-3-glucoside. With methanol as an extraction solvent, a higher content of total xanthone and total anthocyanin can be obtained. The xanthone nanoemulsion composed of soybean oil, Tween 80, CITREM and deionized water, as well as the anthocyanin nanoemulsion composed of ethanol, soybean oil, PEG400, lecithin, Tween 80, glycerol and deionized water, are successfully prepared with high storage and heating stability of both. The IC_50_ of xanthone extract and nanoemulsion in inhibiting HepG2 cells are, respectively, 6.23 and 5.78 μg/mL, while that of the anthocyanin extract is 727.05 μg/mL, with the anthocyanin nanoemulsion showing no inhibition effect. Following the treatment of HepG2 cells with xanthone extract or nanoemulsion, the cell cycle may be arrested at S phase, with the latter inducing a much higher proportion of late apoptosis cells accompanied by a rise in the activities of caspase-3, caspase-8 and caspase-9, leading to cell apoptosis. Taken together, xanthone nanoemulsion showed a more pronounced effect in inducing the apoptosis of HepG2 cells than xanthone extract. 

## Figures and Tables

**Table 1:** Identification data of xanthone and anthocyanin in mangosteen peel extract as well as their contents in dried mangosteen peel extracted by methanol and ethanol.

Peak No.	Compound ^a^	Retention Time (min)		MS/MS (m/z)			Content (μg/g) ^e,f,h^	Content (μg/g) ^e,g,h^
		Standard	Sample	Precursor Ion	Product Ion	Reported		
**Xanthone**								
1	Garcinone C	3.88	3.88	413	297, 283	413, 395, 369, 357, 339, 297, 283, 271 ^b^	513.06 ± 8.25 ^A^	526.12 ± 9.73 ^A^
2	Garcinone D	4.67	4.67	427	353, 412	427, 395, 369, 357, 353, 339, 283 ^b^	469.82 ± 4.68 ^A^	475.38 ± 5.74 ^A^
3	γ-mangostin	5.64	5.64	395	283, 351	395, 283,339,326,297, 351, 271 ^b^	11,100.72 ± 365.36 ^A^	10,984.56 ± 343.27 ^A^
4	8-desoxygartanin	6.25	6.25	379	281, 335	379,363, 335, 321, 281, 269 ^b^	1490.61 ± 15.03 ^A^	1385.45 ± 18.21 ^A^
5	Gartanin	7.96	7.92	395	297, 351	395, 340, 297, 380, 351,337 ^c^	2398.96 ± 34.71 ^A^	2526.34 ± 32.18 ^A^
6	α-mangostin	8.37	8.33	409	351, 377	409, 351, 394, 377, 339 ^b^	51,062.21 ± 838.41 ^A^	50,971.24 ± 440.50 ^A^
7	β-mangostin	12.89	12.89	423	365, 307	423, 408, 391, 365, 353, 307, 295 ^b^	1508.01 ± 5.75 ^A^	1453.39 ± 7.26 ^A^
Total							68,543.39 ± 1257.20 ^A^	68,322.48 ± 856.89 ^A^
**Anthocyanin**								
1	Cyanidin-3- sophoroside	7.25	7.24	611	287, 483	611, 287 ^d^	2889.85 ± 45.65 ^A^	1482.12 ± 30.13 ^B^
2	Cyanidin-3- glucoside	7.67	7.66	449	287, 136	449, 287 ^d^	19.72 ± 0.4 ^A^	16.35 ± 0.61 ^B^
Total							2909.57 ± 46.08 ^A^	1498.47 ± 30.74 ^B^

^a^ Peaks were positively identified based on the comparison of retention time and mass spectra with that reported in the literature. ^b^ Based on a reference by Liang et al. [28]. ^c^ Based on a reference by Wittenauer et al. [29]. ^d^ Based on a reference by Crupi et al. [30]. ^e^ Symbols bearing different capital letter (A–B) in the same row are significantly different at *p* < 0.05. ^f^ Based on extraction with methanol. ^g^ Based on extraction with ethanol. ^h^ Mean of triplicate analyses ± standard deviation.

**Table 2:** Quality control data including recovery, intra-day variability and inter-variability of xanthone and anthocyanin in mangosteen peel as determined by UPLC-MS/MS.

Compound	Recovery						Intra-Day Variability ^c^		Inter-Day Variability ^c^	
	Original (μg)	Spiked (μg)	Found (μg)	Recovery ^a^ (%)	Mean ± SD (%)	RSD ^b^ (%)	Contents (μg/g)	RSD (%) ^b^	Contents (μg/g)	RSD (%) ^b^
**Xanthone**										
Garcinone C	519.06	200	698.06	89.50	91.64 ± 3.02	3.30	503.66 ± 12.74	2.53	516.47 ± 18.96	3.67
		2000	2394.58	93.78						
Garcinone D	467.83	200	656.34	94.25	95.93 ± 2.37	2.47	466.47 ± 6.19	1.33	475.17 ± 9.48	1.99
		2000	2419.92	97.60						
γ-mangostin	11,145.09	4000	14,980.29	95.88	96.68 ± 1.12	1.16	10,715.21 ± 307.69	2.87	11,441.87 ± 237.96	2.08
		40,000	50,133.09	97.47						
8-desoxygartanin	1492.26	400	1878.43	96.54	97.66 ± 1.57	1.61	1474.83 ± 16.79	1.14	1504.75 ± 24.03	1.60
		4000	5443.07	98.77						
Gartanin	2392.29	400	2761.47	92.30	93.98 ± 2.38	2.53	2368.08 ± 61.95	2.62	2436.52 ± 38.09	1.56
		4000	6218.75	95.66						
α-mangostin	51,044.48	5000	55,805.98	95.23	96.54 ± 1.85	1.91	50,232.81 ± 1102.29	2.19	51,909.35 ± 1180.10	2.27
		55,000	104,856.48	97.84						
β-mangostin	1512.00	400	1866.35	88.59	90.34 ± 2.48	2.74	1510.61 ± 14.12	0.93	1501.42 ± 27.69	1.84
		4000	5195.66	92.09						
**Anthocyanin**										
Cyanidin-3- sophoroside	2903.88	500	3362.03	91.63	93.15 ± 2.14	2.30	2838.83 ± 97.40	3.43	2926.84 ± 108.23	3.70
		5000	7636.88	94.66						
Cyanidin-3- glucoside	19.76	10	28.81	90.48	88.37 ± 2.99	3.38	19.27 ± 0.57	2.94	20.12 ± 0.52	2.60
		100	106.01	86.25						

^a^ Recovery (%) = (amount of standard found—original amount in sample)/amount of standard spiked × 100. ^b^ Relative standard deviation (%) = (standard deviation/mean) × 100. ^c^ Mean of triplicate analyses ± standard deviation.

**Table 3:** Particle size, zeta-potential and polydispersity index change as affected by xanthone and anthocyanin nanoemulsion during heating at different temperatures (40–100 °C) for varied length of time (0.5–2.0 h).

Temperature (°C)	Particle Size (nm)				Zeta-Potential (mV)				Polydispersity Index (PDI)			
	0.5 h	1 h	1.5 h	2 h	0.5 h	1 h	1.5 h	2 h	0.5 h	1 h	1.5 h	2 h
**Xanthone Nanoemulsion**												
Control (unheated)	22.4				−89.3				0.229			
40 °C	22.3	22.6	22.7	22.8	−89.1	−89.2	−89.1	−87.5	0.227	0.231	0.236	0.243
60 °C	22.3	22.5	22.8	23.0	−88.9	−88.4	−87.9	−86.9	0.229	0.230	0.243	0.252
80 °C	22.7	22.9	23.1	23.5	−88.0	−87.2	−86.3	−84.9	0.232	0.246	0.254	0.267
100 °C	22.8	23.3	25.8	29.8	−83.6	−81.2	−76.7	−73.0	0.235	0.256	0.279	0.305
**Anthocyanin Nanoemulsion**												
pH = 2												
Control (unheated)	14.2				−39.4				0.153			
40 °C	14.5	15.3	15.9	16.2	−38.9	−37.4	−35.5	−33.6	0.154	0.155	0.153	0.159
60 °C	15.2	15.8	16.3	17.5	−38.5	−36.7	−34.6	−32.7	0.154	0.168	0.183	0.186
80 °C	15.6	16.4	17.6	20.2	−38.1	−35.1	−33.2	−32.2	0.162	0.173	0.199	0.217
100 °C	15.9	18.0	20.2	23.1	−37.0	−34.7	−32.6	−30.7	0.176	0.200	0.231	0.251
pH = 3												
Control (unheated)	14.4				−61.5				0.151			
40 °C	14.4	15.1	15.5	16.1	−60.9	−60.1	−59.3	−58.4	0.148	0.152	0.153	0.151
60 °C	14.6	15.3	16.5	17.2	−60.8	−59.6	−58.5	−57.2	0.153	0.158	0.162	0.164
80 °C	15.1	16.2	16.9	18.8	−59.9	−59.1	−58.4	−57.1	0.161	0.169	0.174	0.185
100 °C	15.2	16.7	18.3	19.8	−58.7	−57.9	−56.2	−53.9	0.162	0.171	0.190	0.206

**Table 4:** Particle size, polydispersity index and zeta-potential of xanthone and anthocyanin nanoemulsion during storage at 4 °C for 90 days.

Storage Time (Day)	Particle Size (nm) ^A^	Polydispersity Index (PDI) ^A^	Zeta Potential (mV) ^A^
**Xanthone Nanoemulsion**			
0	22.0 ± 0.3 ^a^	0.225 ± 0.019 ^a^	−87.7 ± 1.5 ^a^
15	22.5 ± 0.3 ^ab^	0.237 ± 0.014 ^ab^	−87.6 ± 0.4 ^ab^
30	22.1 ± 0.7 ^a^	0.240 ± 0.002 ^ab^	−87.3 ± 0.1 ^ab^
45	22.4 ± 0.3 ^ab^	0.240 ± 0.007 ^ab^	−87.0 ± 0.4 ^ab^
60	22.7 ± 0.2 ^ab^	0.242 ± 0.005 ^ab^	−86.5 ± 0.6 ^ab^
75	22.5 ± 0.3 ^ab^	0.243 ± 0.005 ^ab^	−85.9 ± 1.0 ^b^
90	22.9 ± 0.2 ^b^	0.246 ± 0.011 ^b^	−82.9 ± 1.2 ^c^
**Anthocyanin Nanoemulsion**			
0	14.1 ± 0.5 ^a^	0.148 ± 0.011 ^a^	−61.5 ± 0.7 ^a^
15	14.5 ± 0.2 ^ab^	0.150 ± 0.009 ^a^	−60.1 ± 1.8 ^ab^
30	14.4 ± 0.1 ^ab^	0.155 ± 0.009 ^ab^	−58.8 ± 0.7 ^b^
45	14.6 ± 0.2 ^abc^	0.171 ± 0.008 ^b^	−57.0 ± 0.2 ^c^
60	14.9 ± 0.4 ^bc^	0.189 ± 0.008 ^c^	−55.2 ± 0.4 ^d^
75	15.2 ± 0.3 ^cd^	0.217 ± 0.013 ^d^	−53.3 ± 0.6 ^e^
90	15.8 ± 0.6 ^d^	0.254 ± 0.010 ^e^	−50.7 ± 0.3 ^f^

^A^ Data shown are mean ± standard deviation (n = 3) and data with different small letters (a–f) in the same column are significantly different at *p* < 0.05.

**Table 5:** Effect of different concentrations of xanthone extract and nanoemulsion on cell cycle distribution of liver cancer cell HepG2 ^a^.

Concentration (μg/mL)	Sub-G1 (%)	G0/G1 (%)	S (%)	G2/M (%)
Control	1.10 ± 0.51 ^a^	67.52 ± 0.01 ^a^	8.74 ± 1.16 ^a^	21.76 ± 0.11 ^a^
**Extract**				
6	10.66 ± 0.58 ^b^	56.60 ± 0.16 ^b^	10.22 ± 1.94 ^b^	21.63 ± 2.25 ^a^
8	11.61 ± 0.74 ^c^	56.11 ± 0.41 ^b^	10.47 ± 1.62 ^b^	20.70 ± 1.00 ^a^
10	11.50 ± 0.16 ^c^	53.94 ± 1.33 ^c^	11.82 ± 2.40 ^b^	21.33 ± 1.09 ^a^
**Nanoemulsion**				
6	14.12 ± 0.30 ^d^	50.35 ± 0.39 ^d^	11.44 ± 0.56 ^b^	22.05 ± 1.36 ^a^
8	15.73 ± 0.02 ^e^	48.35 ± 1.61 ^e^	11.85 ± 0.76 ^b^	23.16 ± 1.80 ^a^
10	16.53 ± 0.10 ^f^	47.68 ± 0.28 ^e^	11.95 ± 1.89 ^b^	22.25 ± 1.60 ^a^

^a^ Data shown are mean ± standard deviation (n = 3) and data with different small letters (a–f) in the same column are significantly different at *p* < 0.05.

**Table 6:** Effect of different concentrations of xanthone extract and nanoemulsion on apoptosis of liver cancer cell HepG2 ^a^.

Concentration (μg/mL)	Necrosis Cells A→+) (%)	Late Apoptosis Cells (A+ +) (%)	Viable Cells A→−) (%)	Early Apoptosis Cells (A+ −) (%)
Control	4.19 ± 0.07 ^a^	1.94 ± 0.61 ^a^	88.54 ± 2.84 ^a^	5.36 ± 2.30 ^a^
**Extract**				
6	11.10 ± 1.59 ^b^	27.79 ± 4.21 ^b^	3.86 ± 3.01 ^b^	57.27 ± 0.38 ^b^
8	13.79 ± 3.25 ^c^	30.11 ± 5.49 ^c^	1.70 ± 1.33 ^c^	54.42 ± 0.91 ^c^
10	12.68 ± 3.91 ^d^	54.88 ± 5.71 ^d^	0.40 ± 0.32 ^d^	32.05 ± 1.49 ^d^
**Nanoemulsion**				
6	13.93 ± 4.70 ^e^	48.07 ± 5.89 ^e^	0.15 ± 0.10 ^e^	37.87 ± 1.09 ^e^
8	8.23 ± 3.50 ^f^	81.91 ± 4.69 ^f^	0.07 ± 0.05 ^f^	9.81 ± 1.15 ^f^
10	7.05 ± 2.96 ^g^	85.82 ± 3.77 ^g^	0.05 ± 0.04 ^g^	7.10 ± 0.77 ^g^

^a^ Data shown are mean ± standard deviation (n = 3) and data with different small letters (a–g) in the same column are significantly different at *p* < 0.05.

## Data Availability

The data that support the findings of this study are available within the manuscript.
